# Prolonged dry periods between rainfall events shorten the growth period of the resurrection plant *Reaumuria soongorica*

**DOI:** 10.1002/ece3.3614

**Published:** 2017-12-12

**Authors:** Zhengzhong Zhang, Lishan Shan, Yi Li

**Affiliations:** ^1^ College of Forestry Gansu Agricultural University Lanzhou China

**Keywords:** climate change, desert ecosystems, growth season, rainfall regime, *Reaumuria soongorica*

## Abstract

The resurrection plant *Reaumuria soongorica* is widespread across Asia, southern Europe, and North Africa and is considered to be a constructive keystone species in desert ecosystems, but the impacts of climate change on this species in desert ecosystems are unclear. Here, the morphological responses of *R. soongorica* to changes in rainfall quantity (30% reduction and 30% increase in rainfall quantity) and interval (50% longer drought interval between rainfall events) were tested. Stage‐specific changes in growth were monitored by sampling at the beginning, middle, and end of the growing season. Reduced rainfall decreased the aboveground and total biomass, while additional precipitation generally advanced *R. soongorica* growth and biomass accumulation. An increased interval between rainfall events resulted in an increase in root biomass in the middle of the growing season, followed by a decrease toward the end. The response to the combination of increased rainfall quantity and interval was similar to the response to increased interval alone, suggesting that the effects of changes in rainfall patterns exert a greater influence than increased rainfall quantity. Thus, despite the short duration of this experiment, consequences of changes in rainfall regime on seedling growth were observed. In particular, a prolonged rainfall interval shortened the growth period, suggesting that climate change‐induced rainfall variability may have significant effects on the structure and functioning of desert ecosystems.

## 1 INTRODUCTION

The Badain Jaran Desert climate has changed tremendously over the past 50 years, particularly in terms of rainfall patterns (Ning et al., 2011). Climate change models forecast reductions in annual precipitation and an increased prevalence of extreme events in the region, including longer intervals between rainfall events and fewer rainy days (Yue et al., 2013). Changes in climate affect species in different ways, the consequences of which may not be easy to determine. Previous studies have shown that changes in climate can alter species growth, biomass and morphology (Markesteijn & Poorter, 2009; Root et al., 2003; Wellstein & Cianfaglione, 2014); for example, changes in rainfall were found to cause pronounced mortality in the widespread desert moss *Syntrichia caninervis* (Reed, Coe, & Sparks, 2012). Water is the most important factor limiting the existence and distribution of desert communities. Additionally, some studies have demonstrated that shifts in the rainfall regime (such as changes in timing and duration) might be more important for ecosystems than changes in the quantity of annual rainfall (Reed et al., 2012; Weltzin et al., 2009). Indeed, the effects of climate change on water availability are likely to have widespread consequences for desert ecosystems.

While many plant species are able to survive in arid regions in desert ecosystems, only a few contribute to the majority of the biomass. In the Gobi and Badain Jaran Deserts, the desiccation‐tolerant halophytic desert shrub species *Reaumuria soongorica* (Pall.) Maxim. (Tamaricaceae) is a typical constructive xerophytic species, and it is widespread in desert ecosystems (Zhao, Hai, & Ungar, 2002). In the arid and semiarid Gobi Desert, *R. soongorica* is a constructive and dominant species in the community (Peixi, Chen, & Yan, 2008); it plays an important role in sustaining desert ecological stability (He et al., 2015) and may be considered a keystone species (Ma, Chen, Xia, Wang, & Chen, 2007). Therefore, *R. soongorica* can serve as an indicator of Gobi Desert community trends. This subshrub exhibits various anatomical, morphological, and metabolic adaptations to severe desiccation, including entering a state of dormancy upon dehydration followed by revival when water becomes available (Liu, Wang, et al., 2007). For survival when faced with water deficit, *R. soongorica* starch and nonstructural carbohydrate reserves increase (Xu et al., 2010), and its stems are a crucial organ (Liu, Zhang, Li, & Wang, 2007).

Changes in rainfall are symptoms of climate change. A recent study reported that altered rainfall trends, including declining rainfall quantity and increasing extreme rainfall events, are likely to increase in the future (Yue et al., 2013). Such effects will likely impact the growth characteristics of the species occurring in the Gobi. For example, shrubs showed increased growth with increasing precipitation variability, while grasses showed the opposite response (Gherardi & Sala, 2015a). While drought and decreased rainfall have been found to restrict the production of aboveground biomass (Holub, 2002; Lane, Coffin, & Lauenroth, 2000), the data on belowground biomass are less definitive. Furthermore, the majority of studies have been performed in grasslands, and few data exist with respect to desert ecosystems. Therefore, it is necessary to focus on desert ecosystems, as arid and mesic regions have differential responses to changes in precipitation (Sala, Gherardi, & Peters, 2015).

In this experiment, we tested the effects of changes in rainfall regime on *R. soongorica* growth in the Badain Jaran Desert. Based on the above information, we predicted that changes in the timing of rainfall events are likely to be especially impactful to the growth of *R. soongorica*. With reference to the predicted and observed changes in rainfall in the Badain Jaran Desert, we tested the response of *R. soongorica* growth to the effects of changes in rainfall quantity and timing at three stages during the growth period as well as the interactive effects of changes in rainfall regime and growth period sampling time.

## 2 METHODS

### 2.1 Study site

This study was conducted in a typical Gobi Desert ecosystem located in the southeast of the Badain Jaran Desert (39°24′N, 100°07′E), the third largest desert by area (47,000 km^2^) in China (Dong, Wang, & Wang, 2004). The study site is characterized by a temperate continental climate, the typical features of which are warm summers and long, cold winters. The average annual rainfall is nearly 117 mm and is concentrated largely between June and September, which constitutes the growing season. The annual mean wind speed is 3.2 m/s, and the maximum wind speed is 213.2 m/s. The annual mean air temperature is 7.6°C, while the annual mean surface geotemperature is 13.61°C, and the mean annual evaporation rate over the last 30 years was 2,390 mm (Li, Liu, Liu, Liu, & Niu, 2013). The vegetation of this region is dominated by a few drought‐resistant plants, including *Nitraria sphaerocarpa*,* Salsola passerina*,* Suaeda glauca*, and *Artemisia scoparia*, to name a few. The total plant community cover varies between 6% and 11% (Li et al., 2013).

### 2.2 Experimental design

**Figure 1:**
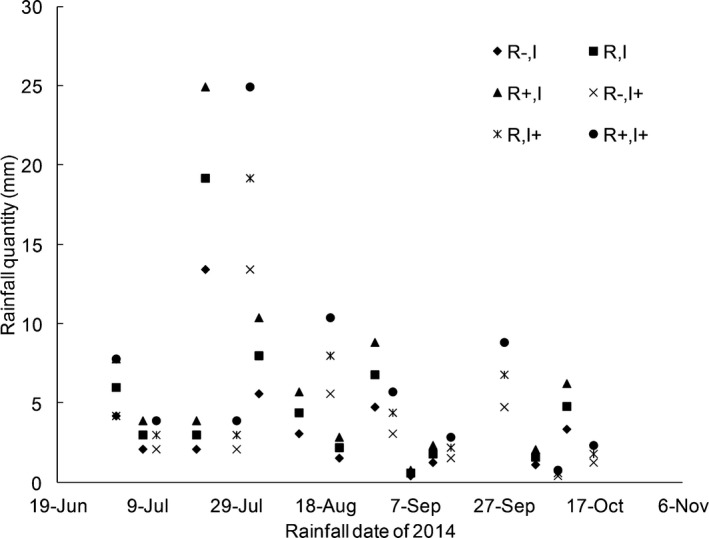
Rainfall quantity and intervals associated with each treatment. *R* indicates the natural rainfall quantity, *R*− indicates 70% of the natural rainfall quantity, and *R*+ indicates 130% of the natural rainfall quantity. It is same as following figures. *I* indicates the natural rainfall interval between two rainfall event, *I*+ indicates 1.5 times of the natural rainfall interval since last rainfall

The experiments began on 1 June 2014. A simple rain gauge was installed at the study site to manually measure the rainfall quantity by collecting rainwater when a rainfall event finished. We adjusted and recorded the rainfall based on the information provided by the device. The experiment included six treatment groups based on two factors: (1) rainfall quantity and (2) interval between rainfall events (Figure 1). We administered water over the top of the plants (similar to rain) using a watering can. The six treatments were as follows:

1. Reduced quantity (*R*−, *I*). Rainout shelters were used to shield the natural rainfall. In this treatment, 70% of the naturally occurring rainfall was immediately applied to the plots (within 12 hr of a rainfall event). This treatment reduced the rainfall quantity without altering the timing of the rainfall events.
2. Natural quantity and interval (*R*,* I*). No rainout shelters were used in this treatment, so no interference to the natural rainfall occurred. The plants were exposed to the naturally occurring rainfall regime, and the rainfall quantity and rainfall time were unaltered.
3. Increased quantity (*R*+, *I*). This treatment also included no rainout shelters, and additional water was applied based on the recorded rainfall quantity. The plants in this treatment were subject to the total naturally occurring rainfall, which was applied immediately, and they were also irrigated to increase the rainfall volume by 30%.
4. Decreased quantity and increased interval (*R*−, *I*+). Rainout shelters were constructed to shield the natural rainfall. The rainfall intervals were lengthened by 50%, and 70% of the natural rainfall quantity was applied in this treatment.
5. Increased interval (*R*,* I*+). Rainout shelters were used to shield the natural rainfall. The rainfall intervals were lengthened by 50%, but water in an amount equivalent to that of natural rainfall was applied.
6. Increased quantity and increased interval (*R*+, *I*+). Rainout shelters were used to shield the natural rainfall. The rainfall intervals were lengthened by 50%, and 130% of the quantity of natural rainfall was applied.

Based on historical rainfall data, the rainfall quantities of the wettest and driest years were within ±30% of the average over the past 50 years, and a 50% increase in the length of the interval between rainfall events can be expected according to the current climate change predictions in this region (IPCC, 2014). For these reasons, we chose to use a 30% alteration in rainfall quantity and 50% increase in the length of the dry period in our experiment. The same parameters have generally been used in previous related studies (Fay, Carlisle, Knapp, Blair, & Collins, 2000; Harper, Blair, Fay, Knapp, & Carlisle, 2005).

### 2.3 Plot construction

Eighteen plots in total were randomly demarcated across approximately 12,000 m^2^ at the study site. Each plot measured 1 × 1 m (this area ensures that more than four 1‐year‐old seedlings can grow without inhibition) and was situated at least 2 m away from the other plots. We ensured that four 1‐year‐old seedlings were growing within every plot and determined that the seedlings were relatively healthy (based on leaf color, crown width, and plant surface integrity). Over 72 seedlings served as controls. Each plot was subjected to one of the six treatments, resulting in three replicates per treatment condition. Each sampled seedling was randomly selected from each plot, and 18 seedlings were harvested at once. As the roots of *R. soongorica* occur mainly in the shallow soil layer, the plots were surrounded by iron straps measuring 50 cm (40 cm beneath the soil, 10 cm above the ground) to eliminate the effects of water infiltration.

A 30‐cm stake was installed in the center of each plot, while a 15‐cm stake was placed in each corner. During rainfall, plastic sheeting (0.1 mm in thickness and 1.4 × 1.4 m in size) was used to construct a rainout shelter. The plastic sheeting was transparent and did not affect illumination. As there was plenty of space under the plastic sheeting, there was no greenhouse effect in the plots. These simple rainout shelters covered 1.3 × 1.3 m in area. The plastic sheeting was only applied when rainfall was expected to occur based on weather forecasts and experience. The treatments *R*,* I* and *R*+, *I* remained unsheltered for the entire duration of the experiment.

### 2.4 Sampling method

To evaluate the effects of changes in rainfall regime on *R. soongorica* seedlings over the growing season, we sampled the plots on 9 August, 17 September, and 22 October 2014, which represented the early, middle, and end of the growing season, respectively. As a rule of thumb, the seedlings adjust to the new environment after being transplanted for approximately 30 days. The first sampling event occurred after the rainfall experienced by the plants had been manipulated for 70 days; thereafter, we ensured that each sampling interval was greater than 30 days to allow the plants to adjust to their respective watering regimes before sampling. In addition, before we started the experiment, a time zero sample was measured to compare conveniently (see Table S1). We sampled the plots three times; during each sampling event, we harvested 18 saplings (1 plant from each plot), with 54 saplings assessed in total. We carefully excavated the complete root system using a traditional method known as the skeleton method (Böhm, 1979). The main root length and plant height were then measured using a tape measure, basal diameter was measured using a vernier caliper, and the plants were transported back to the laboratory. Soil adhering to the seedlings was brushed off, and the aboveground and belowground parts of the plants were separated in preparation for root analysis (total root length, root surface area and root volume) using Epson scanners and WinRHIZO Basic 2008a. The two sections were separately placed in an oven at 105°C for 2 hr and then maintained at 80°C for 48 hr, after which the biomass was weighed using an analytical balance (aboveground biomass and belowground biomass). Root‐shoot ratio and total biomass were calculated based on the aboveground and belowground biomass.

### 2.5 Analyses

We were interested in measuring the effects of changes in rainfall regime and growth period sampling time on biomass and growth indicators. First, the three precipitation treatments (30% increase, natural, 30% decrease) were assessed using one‐way ANOVA, and the two interval treatments (ambient, 50% increase) were also assessed using ANOVA. The analyses were conducted separately for each time period. We focused on plant height, root length, total biomass, aboveground biomass, and belowground biomass for these tests. Then, we assessed the influence of quantity × interval on the treatments using two‐way ANOVA and the same growth indicators as well as root‐shoot ratio, root volume, root surface area, and basal diameter. Additionally, we assessed whether these effects differed across sampling times (early, middle, or late growing season) using three‐way ANOVA (quantity × interval × timing). Bootstrapped *p‐*values were used when the data violated ANOVA assumptions. Tukey's HSD (honest significant difference) tests were used to determine which sample means differed from each other. All analyses were conducted using SPSS 21.0 (Thomey et al., 2011).

## 3 RESULTS

### 3.1 Rainfall regime

In total, 12 rainfall events occurred during the experiment (from July to October), altogether accounting for 64.0 mm of ambient rainfall. The prolonged timing treatment reduced the number of events (10), resulting in a total rainfall quantity of 57.6 mm (Figure 1). Over nearly 50 years, rainfall in the area has exhibited a trend of fluctuating decrease, with the 2014 growing season being drier (64.0 mm) than the average over the last three decades for the same period (96.4 mm). Below‐average rainfall also characterized the growing seasons of 2013 (70.6 mm) and 2015 (60.6 mm).

### 3.2 Morphological changes

**Figure 2:**
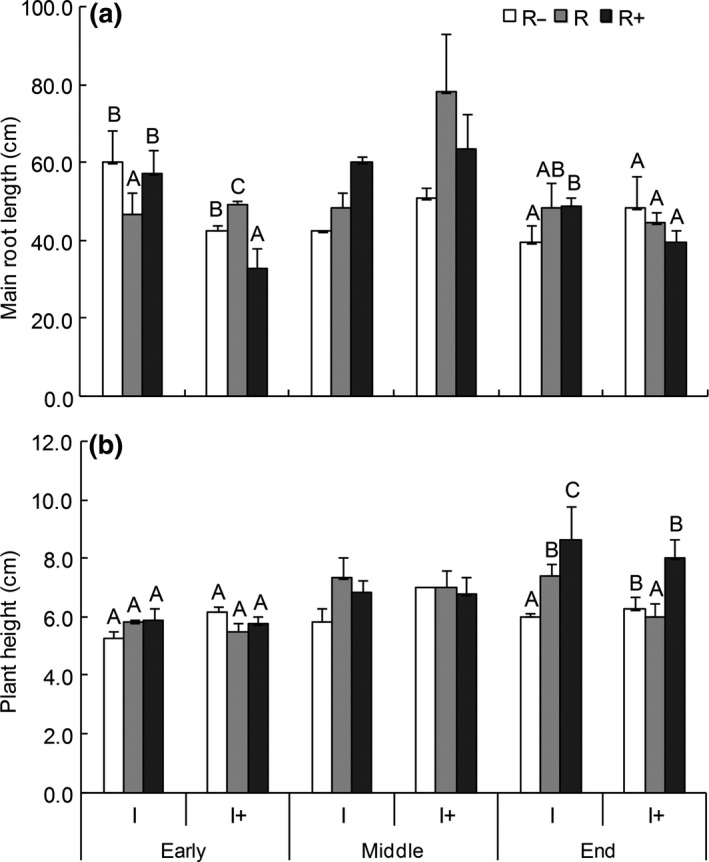
Dynamics of the main root length (a) and plant height (b) of *Reaumuria soongorica* seedlings under different rainfall volumes across the growing season. For all plots, different upper‐case letters are significantly different (*p *<* *.05) based on single‐factor analysis of variance (ANOVA) under the same rainfall interval. Means ± *SE* (*n* = 3)

The one‐way ANOVA showed significant differences in the morphology of *R. soongorica* across the different parts of the growing seasons (Figure 2; Table 1). In general, the increased rainfall interval decreased the main root length and plant height during the early and late stages of the growth period; however, the opposite effect was found during the middle of the growth period. Under the ambient intervals, the main root length and plant height increased significantly with increasing rainfall throughout the entire growth period (Figure 2). However, the different treatments remarkably influenced the main root length and plant growth.

**Table 1:** Results (*F*‐values) based on three‐way ANOVA of the effects of rainfall quantity, rainfall interval, and sampling time on the main root length, plant height, basal diameter, aboveground biomass, belowground biomass, total biomass, and root‐shoot ratio of *Reaumuria soongorica* seedlings

	*Q*	*I*	*T*	*Q* × *I*	*Q* × *T*	*I* × *T*	*Q* × *I* × *T*
Main root length	1.85	0.33	7.87**	4.10*	2.02	7.85***	1.07
Plant height	3.70*	0.08	11.54***	0.64	4.21**	1.26	1.07
Basal diameter	2.42	3.81	3.37*	2.04	1.41	4.92*	1.47
Total root length	0.86	1.70	3.76*	0.88	0.89	5.16*	0.61
Root surface area	0.86	0.96	3.30*	1.61	1.09	4.70*	0.39
Root volume	0.86	0.38	2.59	2.27	1.12	3.34*	0.46
Aboveground biomass	0.85	5.95*	0.41	5.92**	1.94	9.85***	2.84*
Belowground biomass	1.39	1.20	5.66**	1.76	2.04	6.38**	0.96
Root‐shoot ratio	1.31	2.61	3.14	1.00	0.22	3.17	2.46
Total biomass	1.06	4.82*	1.89	4.55*	2.37	9.84***	2.26

The different levels of probability considered are ***p <* *.001, **p <* *.01, *p <* *.05. *Q*, rainfall quantity; *I*, rainfall interval; *T*, the time of sampling. Replicate number = 3.

Changes in interval alone did not significantly affect the morphology of *R. soongorica*, while the growth period significantly impacted several of the morphological indexes. The effect of rainfall quantity (*Q*) × rainfall interval (*I*) on root length was significant, while that of rainfall quantity (*Q*) × sampling time (*T*) significantly affected plant height. The effect of rainfall interval (*I*) × sampling time (*T*) significantly impacted most of the morphological indexes, such as main root length, plant height, basal diameter (Fig. S1), total root length, root surface area (Fig. S2), and root volume (Fig. S3). However, the interaction among the three factors (*Q* × *I* × *T*) had no significant effect on the morphological indexes of *R. soongorica* (Table 1).

The main root length under ambient rainfall quantity and prolonged rainfall duration was highest (78.0 cm), exceeding 70 cm by the middle of the growth season, and the maximum main root length was approached across all treatments by the end of the growth season (Figure 2a). Plant height increased significantly with increased rainfall. At the early stage of the growth season, there were no obvious distinctions in plant height across any of the treatments. However, by the end of the growth season, the heights had all increased, and the differences between most of the treatments were significant (Figure 2b).

### 3.3 Biomass accumulation and allocation

**Figure 3:**
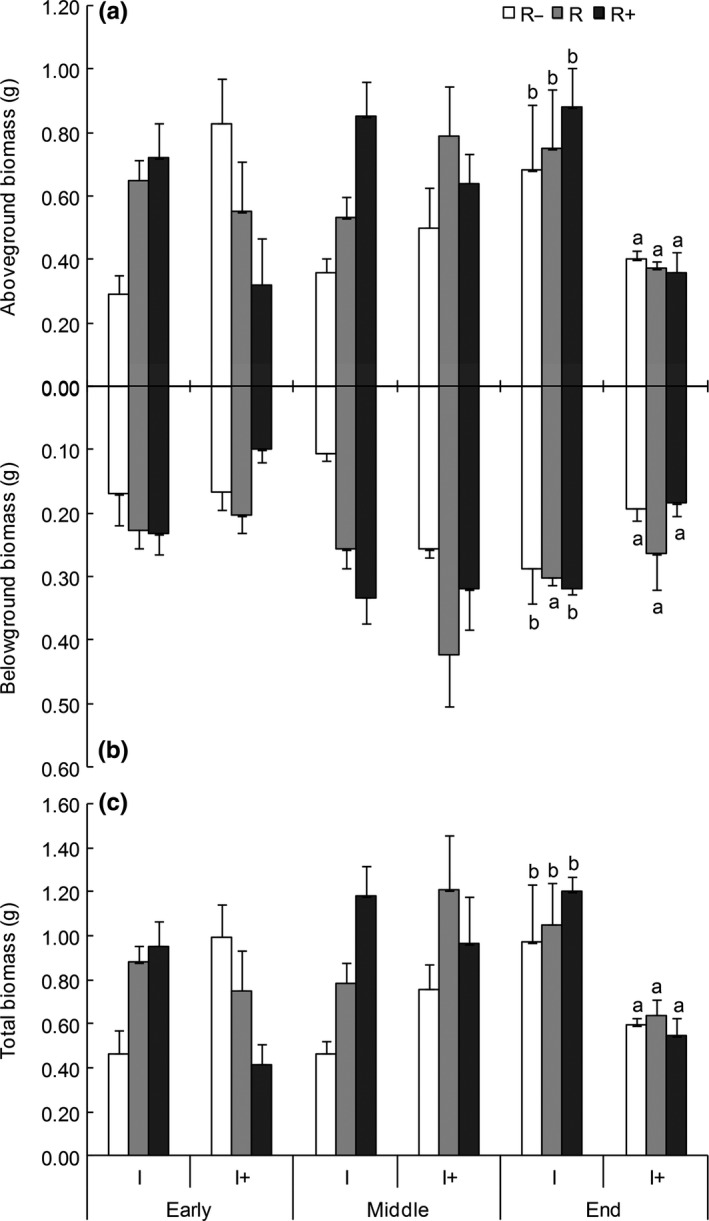
Dynamics of the aboveground biomass (a), belowground biomass (b), total biomass (c) of *Reaumuria soongorica* seedlings under different rainfall volumes across the growing season. For all plots, different lower‐case letters are significantly different (*p *<* *.05) based on single‐factor analysis of variance (ANOVA) under the same rainfall interval. Means ± *SE* (*n* = 3)

All three factors (rainfall quantity, interval and timing) significantly influenced biomass (Table 1). Under ambient rainfall intervals, the aboveground biomass, belowground biomass, and total biomass accumulated with time or increases in rainfall quantity (Table 1, Figure 3). By the middle of the growth season, all indicators of biomass had significantly increased with increasing precipitation under the ambient rainfall intervals. By the end of the growth season, the total biomass and aboveground biomass had decreased significantly only under the prolonged rainfall intervals (Figure 3). Over the entire season, rainfall quantity had no significant effect on aboveground or belowground biomass, while rainfall interval significantly impacted the aboveground and total biomass (*p *<* *.05; Table 1). Underground biomass significantly differed among the sampled growth stages (*p *<* *.01). However, rainfall quantity exerted a greater impact on biomass during the early growth season, while rainfall interval had the greatest effect on biomass in the late growth season (Figure 3).

The rainfall quantity (*Q*) × rainfall interval (*I*) significantly affected the aboveground and total biomass, while rainfall quantity (*Q*) × sampling time (*T*) had no significant effect on biomass. The rainfall interval (*I*) × sampling time (*T*) effect was significant for aboveground, belowground, and total biomass. The interaction among the three factors (*Q* × *I* × *T*) had a significant effect on aboveground biomass. Furthermore, there was no significant effect of each factor alone or interactively on the root‐shoot ratio (Table 1; Fig. S4).

## 4 DISCUSSION

Moisture is the primary limiting factor in arid and semiarid areas, with rainfall constituting the primary water source for plants in these regions. Global climate change is gradually altering rainfall regimes, with a higher frequency of extreme events (including prolonged drought intervals and shorter, more intense rainfall periods) predicted for the future (Diffenbaugh & Giorgi, 2012). Many studies have demonstrated that increased precipitation results in the promotion of growth and the accumulation of biomass, while prolonging the interval between rainfall events has the opposite effect (Ansley, Boutton, & Jacoby, 2014; Fay et al., 2000). Some studies have shown that the interaction between rainfall quantity and rainfall interval is highly variable. However, most studies have involved only sampling once during the growing season and did not assess changes at different stages during the growing season. Furthermore, the majority of studies have focused on grassland ecosystems, while few have examined arid and semiarid environments.

We investigated the effects of changes in rainfall regime (rainfall quantity and interval) on growth and biomass indexes in *R. soongorica* in the early, middle, and late growing season as well as the interactions among these three factors. Water uptake is one of the main root functions (Gregory, 2006), with root systems first experiencing changes in water availability. A study by Steudle (2000) showed that roots and shoots differ in resistance; during water stress, the roots will deliver information to the shoot. Our study shows that the root length greatly differed across treatments during the early growth season but not at the end (Figure 2), while the opposite trend was observed in the stems (plant height). These findings suggest that the root may respond more quickly to rainfall regime changes than the stem. Furthermore, the biomass (aboveground, belowground, and total) greatly decreased when the interval between rainfall events was extended (Figure 3). Therefore, we infer that a prolonged rainfall interval may lead to a shortened growing season. The effects of changes in precipitation quantity and interval on all indexes remarkably differed at the different stages, with the exception of the root‐shoot ratio (Ansley et al., 2014; Holub, 2002).

### 4.1 Additional precipitation advances growth and biomass accumulation

As *R. soongorica* occurs in arid desert regions, it is likely to be sensitive to variation in water availability. Rainfall indirectly affects plant growth by affecting the soil moisture content. Thus, it may be necessary to more deeply study the association between rainfall and soil moisture content in the future studies. Fortunately, the results of previous studies addressing this association have been consistent: Rainfall increases the mean soil moisture content (Hovenden, Newton, & Wills, 2014; Thomey et al., 2011; Wilcox, Fischer, Muscha, Petersen, & Knapp, 2015). In our study, the altered rainfall regime did not change the root‐shoot ratio, and it has been suggested that such results occur because *R. soongorica* is able to adapt to its surroundings (Fay, Kaufman, Nippert, Carlisle, & Harper, 2008; Wu, Zhou, Zheng, Li, & Tang, 2014). However, increased precipitation advanced plant growth and biomass accumulation. Biomass has generally been found to increase along with increased rainfall in arid and semiarid areas, as water stress is the limiting factor in these environments (Kardol et al., 2010; Weiss, Gutzler, Coonrod, & Dahm, 2004), but some studies have suggested that additional precipitation does not influence or may decrease biomass (Fay et al., 2000; White, Campbell, Kemp, & Hunt, 2000). The possible reason for this discrepancy is that the water gradients did not differ enough to reflect the differences.

Under the increased rainfall quantity treatment, the main root length (Figure 2a) and aboveground biomass (Figure 3a) decreased, but the differences were not significant. The small *R. soongorica* seedlings grew better under ambient than increased precipitation, suggesting that this plant is not suited for conditions involving an overabundance of water, as shown in another study that focused more on the effects of precipitation and showed a hump‐shaped response (Gherardi & Sala, 2015b). The effect of the interaction between rainfall quantity and interval was irregular in this study, which may be attributed to the fact that the prolonged rainfall interval altered the growth period.

### 4.2 Growth period is shortened by prolonged rainfall intervals

Recent studies (Hovenden et al., 2014) have emphasized the importance of seasonal rainfall on ecosystems, as rainfall exerts different effects during different seasons. However, many of these studies have used grassland ecosystems as case studies, while studies regarding desert ecosystems are lacking. Furthermore, previous studies have not investigated the effects of differences in rainfall intervals during the growing season. Our study revealed that plants at different growth stages do not respond to prolonged rainfall intervals in the same manner.

Figure 2 indicates that the main root length significantly differed across all treatments in the early growth period, with the differences decreasing toward the end of the growth season, while plant height increased across the different growth periods. Based on these observations, we believe that the roots were better able to respond to the changes in the rainfall regime and adapt to the new environment than the stems (Ropars et al., 2017). As shown in Figure 2a (early season), there appear to be no adaptive response to high rainfall levels. However, it is unsurprising that the roots of this plant rotted under high rainfall conditions, as it is a resurrection plant and is therefore highly adapted to drought conditions. During the prolonged rainfall interval treatments, the biomass measures evaluated in this experiment decreased more significantly at the end of growth season compared to those at the middle of the season (Figure 2). As found by Roux, Mcgeoch, Nyakatya, and Chown (2005), we observed that the seedlings in the prolonged rainfall interval treatments shed their leaves earlier and that sections of the roots had begun to rot (although the plants were still alive). Previous studies (Liu, Wang, et al., 2007) have indicated that *R. soongorica* has a protective mechanism to reduce water loss through leaf abscission in environments lacking water. This reaction occurred under the drought conditions, suggesting that the plants had begun to enter a state of dormancy that would extend until the next rainfall season, thus constituting a strategy to enhance the survival rate during winter. It seems apparent that the growth period had been shortened by the prolonged rainfall interval.

The desiccation‐tolerant *R. soongorica* can survive water contents of more than 5% (Xu et al., 2010) or 15 days of desiccation (Liu, Zhang, et al., 2007). Irregular rainfall patterns stimulate adaptation to the water‐stressed conditions of desert ecosystems. Thus, plant morphology is expected to change in accordance with rainfall during the growing season, as observed in the changes in root length, plant height, aboveground biomass, belowground biomass, and total biomass in this study. While *R. soongorica* was able to persist when the quantity of rainfall was increased or decreased by 30%, it significantly suffered when the dry interval length was extended by 50%. Thus, we believe that the effects of changes in rainfall patterns, such as increased drought intervals, could be more severe than increases or decreases in rainfall on this species. In addition, it is necessary for future studies to clarify the consequences of the priming effect of extreme rainfall and possible lag effects in the plant. Changes in rainfall regime associated with climate change are thus likely to be important factors influencing the response of vegetation in the Gobi Desert. Understanding the interactions among these factors remains a serious challenge for predicting ecosystem responses to climate change.

## CONFLICT OF INTEREST

None declared.

## AUTHOR CONTRIBUTIONS

Zhengzhong Zhang involved in the acquisition, analysis, and interpretation of data for the work, drafting the work, agreement to be accountable for all aspects of the work in ensuring that questions related to the accuracy and integrity of any part of the work are appropriately investigated and resolved. Lishan Shan involved in revising it critically for important intellectual content. Agreement to be accountable for all aspects of the work in ensuring that questions related to the accuracy and integrity of any part of the work are appropriately investigated and resolved, and final approval of the version to be published. Yi Li involved in acquisition of funding. Substantial contributions to the conception and design of the work, revising it critically for important intellectual content, final approval of the version to be published, and agreement to be accountable for all aspects of the work in ensuring that questions related to the accuracy or integrity and any part of the work are appropriately investigated and resolved.
