# Supplementary material for: Prolonged dry periods between rainfall events shorten the growth period of the resurrection plant Reaumuria soongorica
Source: Ecol Evol. 2017 Dec 12;8(2):920–7. doi: 10.1002/ece3.3614 (PMC5773312; doi:10.1002/ece3.3614)
Supplement: Supplementary file 1 [file ECE3-8-920-s001.doc]

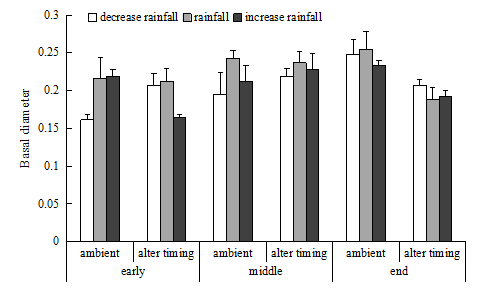


**Fig. S1.** Dynamics of the basal diameter of *Reaumuria soongorica* seedlings under different rainfall volumes across the growing season.


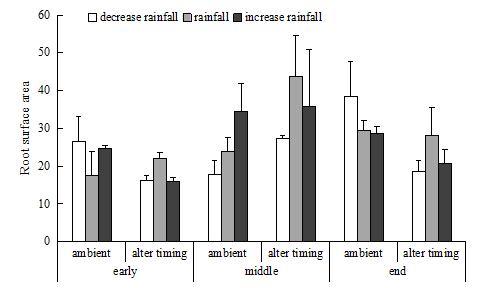


**Fig. S2.** Dynamics of the root surface area of *Reaumuria soongorica* seedlings under different rainfall volumes across the growing season.


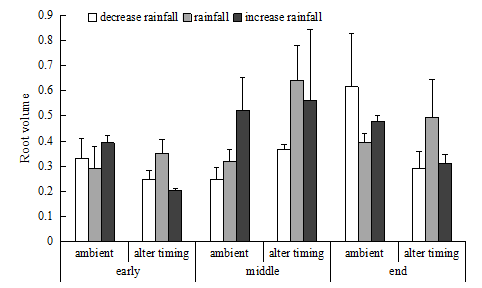


**Fig. S3.** Dynamics of the root volume area of *Reaumuria soongorica* seedlings under different rainfall volumes across the growing season.


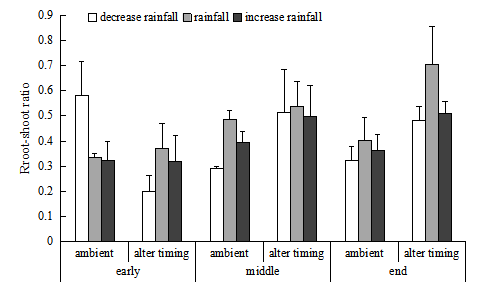


**Fig. S4.** Dynamics of the root-shoot ratio of *Reaumuria soongorica* seedlings under different rainfall volumes across the growing season.

**Table S1.** The condition of *Reaumuria soongorica* before the control. Means ± SE (n=3)

| Main root length (cm) | Plant height (cm) | Basal diameter (mm) | Total root length (cm) | Root surface area (cm^2^) | Root Volume (cm^3^) | Aboveground biomass (g) | Belowground biomass (g) |
| --- | --- | --- | --- | --- | --- | --- | --- |
| 15.4±0.6 | 1.33±0.12 | 8.2±0.76 | 40.40±0.31 | 4.13±0.44 | 0.045±0.003 | 0.1151±0.0212 | 0.0838±0.0018 |

The data measured on June 1,2014.
